# Pharmacokinetics, Pharmacodynamic Efficacy Prediction Indexes and Monte Carlo Simulations of Enrofloxacin Hydrochloride Against Bacterial Strains That Induce Common Clinical Diseases in Broiler Chickens

**DOI:** 10.3389/fvets.2020.606872

**Published:** 2021-01-07

**Authors:** Karina P. D. Bonassa, Miwa Y. Miragliotta, Rosineide C. Simas, Marcos N. Eberlin, Arturo Anadón, Ronilson A. Moreno, Felix G. R. Reyes

**Affiliations:** ^1^Department of Food Science, School of Food Engineering, University of Campinas, Campinas, Brazil; ^2^AGRIAS Pesquisa & Desenvolvimento S.A.R.C. no Agronegócio Ltda., Amparo, Brazil; ^3^Laboratory of Chromatography and Mass Spectrometry, Institute of Chemistry, Federal University of Goias, Goiania, Brazil; ^4^MackMass Laboratory for Mass Spectrometry, School of Engineering, Mackenzie Presbyterian University, São Paulo, Brazil; ^5^Department of Pharmacology and Toxicology, Faculty of Veterinary Medicine, Universidad Complutense de Madrid, Madrid, Spain

**Keywords:** enrofloxacin hydrochloride, ciprofloxacin, pharmacokinetics, pharmacodynamics, MIC values, PK/PD modeling, Monte Carlo simulations, broiler chicken

## Abstract

Pharmacokinetic parameters and efficacy prediction indexes (C_max_/MIC_90_ and AUC_0−24_/MIC_90_) of an enrofloxacin hydrochloride (ENR-HCl) veterinary product soluble in water were determined in healthy broiler chickens of both sexes after a single oral dose of ENR-HCl (equivalent to 10 mg ENR base/kg bw). Monte Carlo simulations targeting C_max_/MIC_90_ = 10 and AUC_0−24_/MIC_90_ =125 were also performed based on a set of MIC (minimum inhibitory concentration) values of bacterial strains that induce common clinical diseases in broiler chickens and that showed to be susceptible to ENR-HCl. Plasma concentrations of ENR and its main metabolite ciprofloxacin (CIP) were determined by liquid chromatography-tandem mass spectrometry (LC-MS/MS). Plasma concentration-time curves were found to fit a non-compartmental open model. The ratio of the area under the plasma concentration-time curve (AUC) of CIP/ENR was 4.91%. Maximum plasma concentrations of 1.35 ± 0.15 μg/mL for ENR-HCl and 0.09 ± 0.01 μg/mL for CIP were reached at 4.00 ± 0.00 h and 3.44 ± 1.01 h, respectively. Areas under the plasma vs. time concentration curve in 24 h (AUC_0−24_) were 18.91 ± 1.91 h × μg/mL and 1.19 ± 0.12 h × μg/mL for ENR-HCl and CIP, respectively. Using a microbroth dilution method, the minimum inhibitory concentration (MIC_90_) values were determined for ENR-HCl for 10 bacterial strains (*Mycoplasma gallisepticum, Mycoplasma synoviae, Avibacterium paragallinarum, Clostridium perfringens, Escherichia coli, Pseudomonas aeruginosa, Salmonella* ser. Enteritidis*, Salmonella* ser. Gallinarum*, Salmonella* ser. Pullorum, and *Salmonella* ser. Typhimurium), which are the most common causes of infectious clinical diseases in broiler chickens. In summary, the PK/PD ratios and Monte Carlo simulation were carried out for ENR-HCl in poultry, which due to its solubility was administered in drinking water. The PK/PD efficacy prediction indexes and Monte Carlo simulations indicated that the ENR-HCl oral dose used in this study is useful for bacterial infections in treating *C. perfringens* (Gram-positive), *E. coli* and *S*. ser. Enteritidis (Gram-negative) and *M. gallisepticum* bacteria responsible for systemic infections in poultry, predicting a success rate of 100% when MIC ≤ 0.06 μg/mL for *E. coli* and *S*. ser. Enteritidis and MIC ≤ 0.1 μg/mL for *M. gallisepticum*. For *C. perfringens*, the success rate was 98.26% for MIC ≤ 0.12. However, clinical trials are needed to confirm this recommendation.

## Introduction

Fluoroquinolones (FQs) were developed to overcome the limitations exhibited by several antibacterial agents. Their broad spectrum of antibacterial activity, good oral availability and distribution in tissues, longer elimination half-life and considerably reduced toxicity make FQs the preferred antimicrobial agent for treating a broad variety of bacterial infections, not only in veterinary use but also in humans (1).

Enrofloxacin hydrochloride (ENR-HCl) (1-cyclopropyl-7-(4-ethylpiperazin-1-yl) -6-fluoro-4-oxoquinoline-3-carboxylic acid; hydrochloride; CAS No. 112732-17-9) is a FQ exclusively used in veterinary medicine that is metabolized by the liver into ciprofloxacin (CIP), which is an equipotent metabolite (2). ENR has high activity against Gram-negative and Gram-positive bacteria and *Mycoplasma* spp. (3, 4). ENR acts in DNA synthesis by inhibiting two topoisomerases that are essential to bacterial replication, including DNA gyrase, which induces the supercoiling of the chromosome, and topoisomerase IV, which helps divide replicated chromosomes (5).

The potential usefulness of ENR-HCl as an antibacterial agent for the treatment of common infections in poultry requires detailed information on PK/PD indices and the optimum level of drug exposure associated with the susceptibility of the agent to thereby limit the development of resistance in target pathogens (6). Furthermore, ENR must not be used for prophylaxis treatment or when resistance/cross-resistance to this veterinary drug is known to occur in the flock intended for treatment. The use of ENR as a critically important antimicrobial (CIA) should be limited to cases where no other alternative is available. Whenever possible, ENR should only be used based on culture and antimicrobial susceptibility testing (C&AST).

ENR at low doses does not guarantee efficacy, as it is considered a concentration-dependent bactericidal antibiotic, with a dose-response curve that is characterized by low activity or a loss of activity. Thus, in relation to the most important factors for predicting efficacy, i.e., a maximum plasma concentration (C_max_)/minimum inhibitory concentration (MIC) ratio (C_max_/MIC_90_) and an area under the curve (AUC) at 24 h/MIC_90_ ratio (AUC_0−24_/MIC_90_) it was suggested that the AUC:MIC required for successful quinolone therapy of infection due to Gram-negative bacteria appears to be higher (~100) (7). Furthermore, recent PK/PD studies conducted with ENRO- HCl-2H_2_O on dogs (8) and cows (9) against *Leptospira* spp. reported Monte Carlo simulations based on target attainment ratios of *C*_max_/MIC_90_ = 10 and AUC_0−24_/MIC_90_ = 125, as functions of optimal serum bactericidal concentration. Moreover, the plasma concentration of an antimicrobial drug does not necessarily reflect its capacity to penetrate the location of the bacterial infection (“biophase”) or the inside of the bacterial cell, since other factors can also modify the bacterial activity of an antimicrobial, such as the intracellular pH relationship, the drug pKa, the oxygen content and the intracellular enzymatic activity.

ENR is rapidly absorbed following oral administration, achieving steady-state serum concentrations that are in excess of the MIC values for most pathogens. The oral absorption of ENR is rapid, with peak serum concentrations achieved 1–2 h after administration, making it a highly effective bactericidal compound with relatively low MIC values (10). Usually, the behavior of ENR has a flatter time-concentration curve, lower peak concentrations, and a longer elimination half-life compared to other antimicrobial agents. The low C_max_ of ENR may be a consequence of the low plasmatic protein binding and high tissue uptake. ENR has a high volume of distribution and penetration in tissues, and this contributes to a lower excretion and a longer elimination half-life (4, 11, 12).

Studies of the relationship between antimicrobial treatment and the risk of selection of resistance in both human and animal subjects show interrelationships among the drug concentration, the duration of exposure, and the bacterial load (13).

Studies have been published regarding ENR base in avian pharmacokinetics. For instance, in chickens (4, 14–18) and turkeys (3, 19). In addition, Gutierrez et al. (20) reported notable improvements in the main pharmacokinetic parameters (PK) and in PK/PD indices in broiler chickens after oral administration of ENR-HCl dihydrate compared to ENR base. The authors also reported that considering efficacy prediction indexes (C_max_/MIC and AUC/MIC) and the minimal inhibitory concentration (MIC) values for wild-type *Escherichia coli* O78/H12, optimal ratios will only be achieved by ENR-HCl dihydrate. However, as far as we know, there are no previous reports describing the utilization of ENR-HCl to treat pathogenic strains responsible for the common bacterial diseases in broiler chickens, as we report in this study.

Normally, ENR base, destined for broiler chickens, is prepared in solution in a strongly alkaline vehicle, usually KOH, and administered through drinking water, which has shown limited bioavailability (~60%) in these species and low solubility of the active substance in gastrointestinal transit (14, 21, 22). However, ENR-HCl dyhydrate showed greater oral bioavailability in chickens than its reference preparation (Bayer® Animal Health S.A de C.V., Mexico City) (20). Thus, considering (a) that the development of new pharmaceutical products is a route to provide different, more accessible and higher efficacy veterinary medicinal products for threatening diseases in food animals, and (b) the fact that bacterial diseases remain a relevant issue in production systems in both developed and developing countries, the aim of this study was to evaluate an ENR-HCl pharmaceutical product soluble in water to assess its oral effectiveness against 10 bacterial strains responsible for the most common causes of infectious clinical diseases in broiler chickens. For this purpose, the pharmacokinetic parameters of an oral dose of ENR-HCl (equivalent to 10 mg ENR base/kg bw) of the product were determined, the pharmacodynamic efficacy prediction indexes (C_max_/MIC_90_ and AUC_0−24_/MIC_90_) were estimated, and Monte Carlo simulations (utilizing *in vitro* data of the bacterial strain susceptibility to ENR) were conducted as predictors of the potential clinical efficacy of the ENR-HCl product in treating infections.

## Materials and Methods

### Chemicals and Reagents

Analytical standards of ENR base (higher than 98% purity) and CIP (higher than 98% purity) were purchased from Dr. Ehrenstorfer GmbH (Augsburg, Germany), and CIP-*d8* hydrochloride hydrate (internal standard; IS) was purchased from Sigma-Aldrich (St. Louis, MO, USA). Solid phase extraction (SPE) Strata-X cartridges (polymeric reversed-phase, 60 mg/3 mL) were purchased from Phenomenex (Torrance, CA, USA). All chromatographic solvents used in this study were HPLC grade. All other chemical reagents used were obtained from usual commercial sources and were of analytical grade. Water was first purified by distillation and then passed through a Milli-Q system (Millipore, Bedford, MA, USA).

### Veterinary Pharmaceutical Product Formulation

In this study, a veterinary pharmaceutical product that contains a nominal concentration of 25% ENR-HCl was used. The ENR-HCl (≥99% purity) batch number DK05-1306071 was supplied by Shangyu Jingxin Pharmaceutical Co., Ltd. (Shangyu, China). This veterinary pharmaceutical product is intended to be commercialized in powder to be diluted in drinking water for administration to broiler chickens. Prior to use in this study, the real concentration of ENR base in the product formulation was determined by a validated HPLC-UV detector analytical method (23).

### Animals

This study was developed in accordance with the ethics requirements and was authorized by the official ethical committee of the University of Campinas (Protocol number 3135-1). Twelve clinically healthy male and female broiler chickens (commercial *Gallus gallus domesticus*) (Cobb®) were used in equal proportions. All chickens were 41 days old (1.95–2.79 kg body weight), in good health, and obtained from a poultry breeding farm (Agroceres, Patrocínio, State of Minas Gerais, Brazil). The chickens were placed in experimental one-floor pens and were maintained for an 8-day acclimation period prior to the study. The environmental conditions were maintained at 25°C ± 5°C with 50–60% relative humidity, and the animals had *ad libitum* access to water. The feed provided was formulated according to the nutrient requirements for broiler chickens being grown for market and was free of antimicrobial drugs.

### Experimental Design

After the acclimation period of 8 days, the chickens were weighed and nine chickens (five males and four females) were randomly preselected for the study (three were reserved for use as substitutes if necessary). The veterinary pharmaceutical product was dissolved in sterile distilled water. This solution (treatment solution) contained 4 mg ENR-base/mL. Thus, a volume of the treatment solution was individually calculated for each chicken to provide a dose equivalent to 10 mg ENR-base/kg bw. The treatment solution was administered directly into the crop (“*gavage*”) using a thin plastic tube attached to a syringe to ensure complete ingestion of the dose. Food, but not water, was withheld for 12 h before dosing and until 6 h after drug administration. All dosages were given between 8 a.m. and 9 a.m.

Blood samples (1.5 mL) were taken from the *brachialis vein* from each of the nine chickens at each time point. The samples were collected in tubes containing an anticoagulant (EDTA) prior to treatment and at 0.5, 1, 2, 3, 4, 6, 8, 12, and 24 h after drug administration. The blood samples were centrifuged (3,000 × *g* for 10 min), and the plasma was stored at −50°C until LC-MS/MS analysis.

### Determination of Enrofloxacin and Ciprofloxacin Concentrations in Plasma

ENR and CIP concentrations in the plasma were determined by LC-MS/MS, as reported by Ferrari et al. (24). The developed LC-MS/MS analytical method was fully validated according to EU requirements (25) and showed the following validation parameters: the linear range of the analytical curves was 1–150 ng/mL with a correlation coefficient (*R*^2^) exceeding 0.998 for both ENR and CIP. The intra- and inter-day recoveries were determined at four concentration levels, and they ranged from 93% to 111% (ENR) and 102 to 113% (CIP). Overall, the intra- and inter-day precision for ENR and CIP expressed as the coefficient variation percentage (CV%), ranged from 1.33 to 10.9%. The method limit of quantitation (LOQ) was 1.0 ng/mL for both ENR and CIP. Interference of endogenous compounds (matrix effect) was verified for ENR (7–17%) and CIP (6–16%) using blank plasma from untreated chickens. Therefore, since quantitation using matrix-matched analytical curves is recommended when the matrix effect is observed, the analytical curves were prepared from blank plasma samples spiked with the appropriate standard solutions as described by Ferrari et al. (24), and drug concentrations were determined using the matrix-matched analytical curves. To quantify ENR in plasma, the clean extracts obtained were properly diluted before injection into the LC-MS/MS system in order to have a detection response within the linear range of the analytical curves.

### Pharmacokinetic Parameters and Monte Carlo Simulations

The plasma concentration vs. time data fit a non-compartmental model using WinNonlin 6.4 (Pharsight Corporation, Mountain View, CA, USA). The best fit was determined according to Akaike's Information Criterion. The area under the plasma concentration-time curve (AUC) was calculated using the linear trapezoidal rule between time 0 and 24 h and extrapolating the area to infinity by means of the elimination rate constant (Equation 1).

(1) $$\begin{array}{r} AUC_{0-t_{k}}=\sum_{i=1}^{k}\left(\frac{C_{i-1}+C_{i}}{2}\right)\left(t_{i}-t_{i-1}\right) \end{array}$$

C_max_ and the time to reach C_max_ (T_max_) were determined directly from the concentration vs. time curve.

The terminal elimination rate constant (Ke) was calculated from the log-linear portion of the elimination curve using a linear regression analysis

The elimination half-life (t_1/2_ e) was calculated according to Equation 2:

(2) $$\begin{array}{r} t_{1/2}e=\left(\ln\;2\right)/\text{Ke}. \end{array}$$

The area under the first moment curve (AUMC) was calculated according to Equation 3:

(3) $$\begin{array}{r} AUMC_{0-t_{k}}=\sum_{i=1}^{k}\left(\frac{{ti.\;C}_{i\;+ti+1\cdot }C_{i+1}}{2}\right)\left(t_{i}-t_{i+1}\right) \end{array}$$

The mean residence time (MRT) was calculated according to Equation 4:

(4) $$\begin{array}{r} \text{MRT}=\text{AUMC}/\text{AUC} \end{array}$$

Monte Carlo simulations were performed using GraphPad Prism version 6.00 for Windows (GraphPad Software, La Jolla, California, USA). Taking into account key PK data (C_max_ and AUC_0−24_ parameters) and efficacy prediction index (PK/PD index), bacteria susceptible to ENR-HCl (*E. coli, M. gallisepticum, C. perfringens, ATCC*, and *S*. ser. Enteritidis) were subjected to Monte Carlo simulations considering a normal distribution, based on the target attainments of C_max_/MIC_90_ = 10 and AUC_0−24_/MIC_90_ = 125, as functions of optimal plasma bactericidal concentration, simulating 10,000 individuals. MIC_90_ values included those determined in this study, as well as a set of reported MIC values of the sensitive bacterial population for each of the bacteria strain. The probability of target attainment is expressed as the percentage of the population reaching or exceeding the specific target. The sum of ENR and CIP concentrations was used for the PK/PD integration and modeling.

### Bacterial Strains and Antimicrobial Susceptibility Testing

A total of 10 bacterial strains, representing the most common causes of disease in chickens, were used. Among them, *Avibacterium paragallinarum, Salmonella* ser. *Enteritidis, Salmonella* ser. *Gallinarum, Salmonella* ser. *Pullorum*, and *Salmonella* ser. Typhimurium strains from the CEDISA laboratory (Animal Health Diagnostic Center) were isolated from broiler chickens found in clinical cases of diseases on farms. *Clostridium perfringens ATCC 13124, Escherichia coli ATCC 25922, Mycoplasma gallisepticum* ATCC 15302, *Mycoplasma synoviae* ATCC 25204, and *Pseudomonas aeruginosa ATCC 27853* were obtained from the American Type Culture Collection (ATCC, Rockville, Maryland, USA).

The ENR-HCl veterinary product formulation was dissolved in water at a concentration range between 0.004 and 512 μg/mL. The minimum inhibitory concentration (MIC_90_) was determined by the microbroth dilution method (26). *Staphylococcus aureus* ATCC 29213 was used for quality control in the MIC determinations. The breakpoint values established by CLSI (26) were used, and some were obtained from the available literature.

## Results

### Plasma Enrofloxacin Disposition After a Single Oral Administration of the Product Formulation Containing Enrofloxacin Hydrochloride

**Figure 1:**
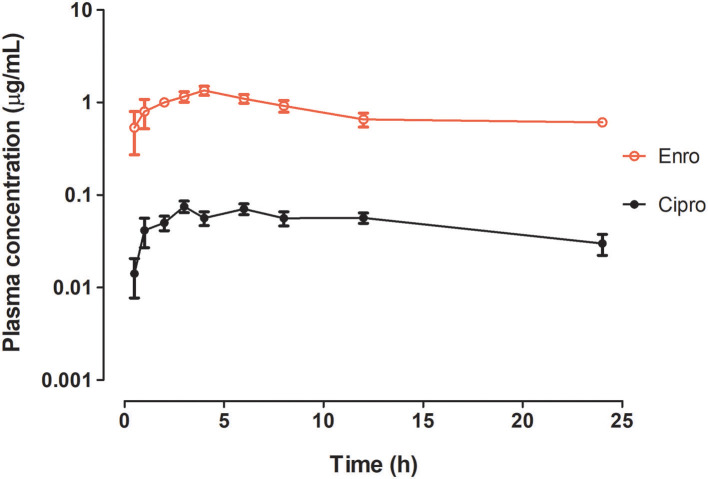
Plot of the plasma concentration (mean ± SD) of enrofloxacin (ENR) and its active metabolite ciprofloxacin (CIP) in broiler chickens following single oral administration of a veterinary pharmaceutical formulation that contains ENR-HCl. Dose administered equivalent to 10 mg ENR base/kg bw.

Mean plasma concentrations (μg/mL ± SD) of ENR and CIP after oral administration (“*gavage*”) of the veterinary product formulation containing ENR-HCl are shown in Figure 1, and the plasma pharmacokinetic parameters are presented in Table 1. In this study, the C_max_ for ENR-HCl in plasma was 1.35 μg/mL, and the time taken to reach T_max_ was 4 h. The level of CIP in the plasma was 4.9%, as calculated by the ratio between the mean AUC_0−∞_ for CIP and the mean AUC_0−∞_ for ENR-HCl. It was verified that there was a slow elimination phase for ENR-HCl and CIP, which was characterized by a half-life of elimination of 24.76 ± 3.67 h and 17.45 ± 6.40 h for ENR-HCl and CIP, respectively.

**Table 1:** Enrofloxacin and its metabolite ciprofloxacin plasma pharmacokinetic parameters (mean ± SD, *n* = 9) for broiler chickens after a single oral dose (10 mg/kg bw) administration of a new veterinary pharmaceutical formulation that contains enrofloxacin hydrochloride.

**Parameters**	**Enrofloxacin**	**Ciprofloxacin**
C_max_ (μg/mL)	1.35 ± 0.15	0.08 ± 0.01
T_max_ (h)	4.00 ± 0.00	3.44 ± 1.01
K_e_ (h^−1^)	0.03 ± 0.00	0.04 ± 0.01
t_1/2_ e (h)	24.76 ± 3.67	17.46 ± 6.40
AUC_0−24_ (h × μg/mL)	18.91 ± 1.91	1.19 ± 0.12
AUC_0−∞_ (h × μg/mL)	40.73 ± 5.18	2.00 ± 0.61
AUMC_0−∞_ (h^2^ × μg/mL)	200.28 ± 20.50	12.64 ± 1.63
MRT (h)	10.60 ± 0.32	10.59 ± 0.42

*C_max_, maximal plasma concentration; T_max_, time needed to reach C_max_; K_e_, elimination rate constant; t_1/2_ e, half-life time of elimination phase; AUC_0−24_, area under the concentration vs. time curve from 0 to 24 h; AUC_0−∞_, area under the concentration vs. time curve; AUMC_0−∞_, area under the first moment curve*.

*MRT, mean residence time*.

### Efficacy Predictors

The estimated values of the ratios of C_max_/MIC_90_ and AUC_0−24_/MIC_90_ are shown in Table 2. The applied C_max_ and AUC_0−24_ values were 1.35 μg/mL and 18.91 h × μ/mL for ENR-HCl and 0.08 μg/mL and 1.19 h × μ/mL for CIP, respectively.

**Table 2:** Efficacy prediction indexes (C_max_/MIC_90_ and AUC_0−24_/MIC_90_) estimated for enrofloxacin against susceptible bacteria in broiler chickens after a single oral dose (10 mg/kg bw) administration of a new veterinary pharmaceutical formulation that contains enrofloxacin hydrochloride.

		**Predictors**					
		**C_**MAX**_/MIC_**90**_**	**AUC_**0−24**_/MIC_**90**_ (h)**	**C_**MAX**_/MIC_**90**_**	**AUC_**0−24**_/MIC_**90**_ (h)**	**C_**MAX**_/MIC_**90**_**	**AUC_**0−24**_/MIC_**90**_ (h)**
**Bacteria**	**MIC_**90**_ (μg/mL^**)**^**	**Enrofloxacin**		**Ciprofloxacin**		**ENR** **+** **CIP**	
*Mycoplasma gallisepticum*	0.016	84	1,182	5	74	89	1,256
*Mycoplasma synoviae*	0.5	3	38	0	2	3	40
*Escherichia coli*	0.004	336	4,726	20	298	356	5,024
*Avibacterium paragallinarum*	16	0	1	0	0	0	1
*Clostridium perfringens*	0.125	11	151	1	10	12	161
*Pseudomonas aeruginosa*	0.5	3	38	0	2	3	40
*Salmonella* ser. Enteritidis	0.008	168	2,363	10	149	178	2,512
*Salmonella* ser. Gallinarum	2	1	9	0	1	1	10
*Salmonella* ser. Pullorum	0.5	3	38	0	2	3	40
*Salmonella* ser. Typhimurium	0.5	3	38	0	2	3	40

**Figure 2:**
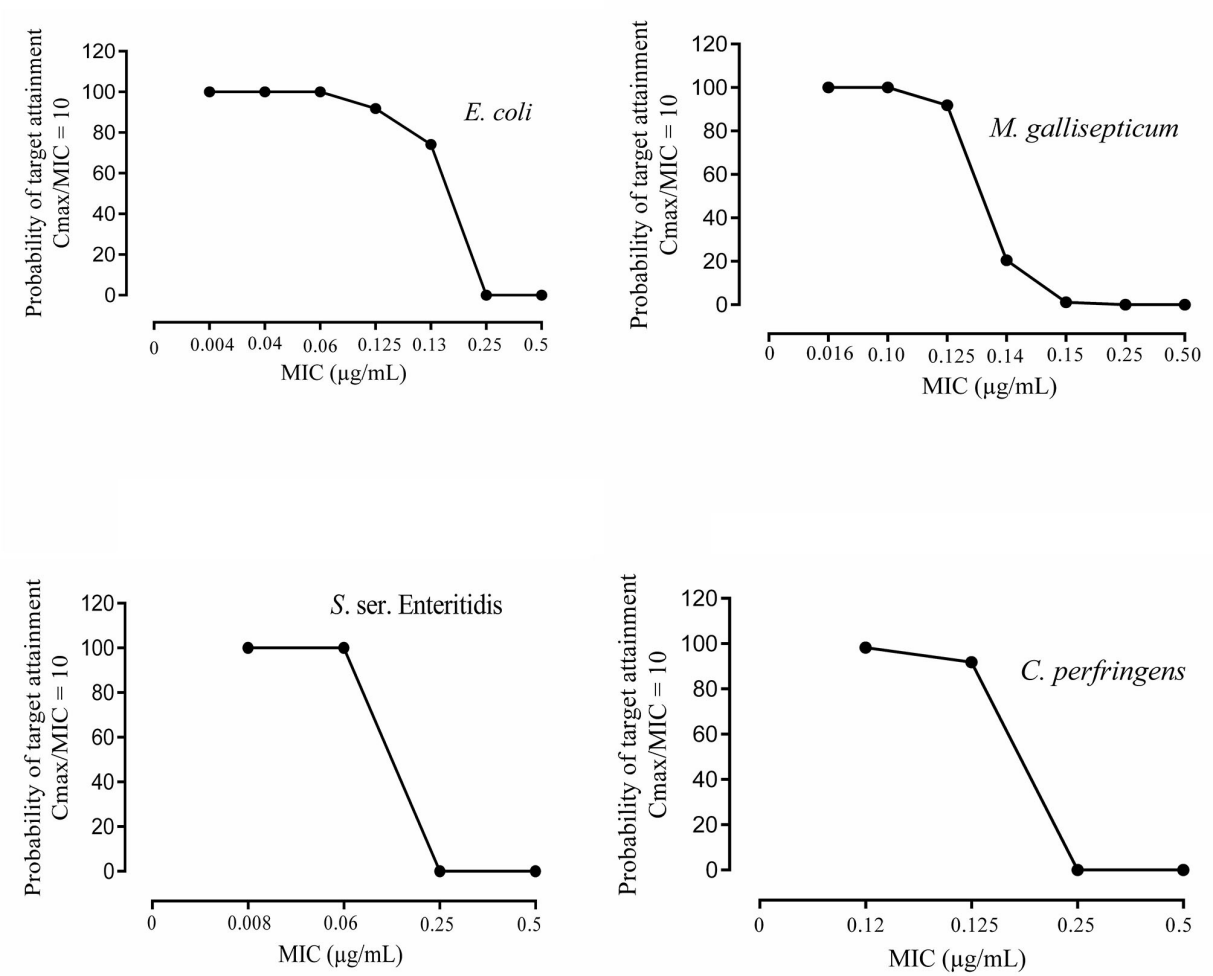
Probability of target attainment (Cmax/MIC_90_ = 10) of the bacteria susceptible to ENR-HCl veterinary product formulation after a single oral dose (equivalent to 10 mg ENR base/kg bw) vs. a survey of minimum inhibitory concentration (MIC_90_) values. The MIC_90_ values determined in this study for each of the bacteria strains were also included. C_max_, maximum plasma concentration.

The results of the Monte Carlo simulation are shown in Figure 2, where the probability of reaching the target attainment C_max_/MIC_90_ = 10 for the ENR-HCl product formulation, oral route at a dose equivalent to 10 mg ENR base/kg bw, is presented against the MIC values for each of the strains that induce common clinical diseases in broiler chickens. The results related to AUC_0−24_/MIC_90_ = 125 were similar of what is exhibited in Figure 2 and for this reason, they are not shown. However, although Monte Carlo simulation results of the target attainments were the same for *S*. ser. *Enteritidis* and *C. perfringens*, there was a small difference between *E. coli* and *M. gallisepticum*. At MIC value of 0.125, both *E. coli* and *M. gallisepticum* showed a TAR value of 100 and 91.84% for AUC_0−24_/MIC = 125 and C_max_/MIC = 10, respectively.

## Discussion

ENR is still an option for treating food-producing avian farmed in Africa, Asia, some parts of Europe and Latin America countries. Moreover, the lack of bioequivalence in generic formulations may be a relevant issue in terms of antimicrobial efficacy (20, 27, 28). Furthermore, PK/PD predictive indices of *in vivo* efficacy are often not obtained, resulting in a logical increase in bacterial resistance and lack of efficacy in the treatment of clinical infectious diseases (18). In this study we adopted the most common ratio of C_max_/MIC_90_ = 10 and AUC_0−24_/MIC = 125 (8, 9). It is important to consider the fact that an efficient treatment using an antimicrobial essentially depends on the relationship between the precise diagnosis and the adequate administration of the antibacterial agent, and for that reason, it must evaluate and respect the information on the ideal dosage regime to use the veterinary drug safely to reach a clinical cure (29).

Plasma disposition of ENR and CIP in broiler chickens using a non-compartmental model and a single oral administration of the veterinary product containing ENR-HCl (dose equivalent to 10 mg ENR base/kg bw) is shown in Table 1. C_max_ values of 1.35 and 0.08 μg/mL were obtained for ENR-HCl and CIP, respectively, which were similar to the values reported for ENR-HCl in turkeys (1.23 and 0.08 μg/mL for ENR and CIP, respectively) (3) and for ENR base in broiler chickens by Silva et al. (2) (1.5 μg/mL). Notwithstanding, for ENR-HCl dihydrate in broiler chickens, a C_max_ of 5.90 μg/mL was reported by Gutierrez et al. (20). In addition, for the ENR base, Anadón et al. (14) reported an ENR C_max_ value of 2.44 μg/mL, and for a reference and three ENR generic preparations, all formulated as water-soluble products, Sumano et al. (27) reported values in the range of 0.99–3.05 μg/mL. Additionally, for a dose of 20 mg ENR base/kg bw, Gberindyer et al. (16) reported values in the range of 0.69–1.00 μg/mL for three generic oral formulation products from different pharmaceutical companies Therefore, data indicate that the C_max_ values depend on the pharmacologically active substance form and on the pharmaceutical product formulation that is administered to the chickens, i.e., the drug (ENR base, ENR-HCl or ENR-HCl dihydrate), rather than on the polarity of the active substance. Other factors that could contribute to C_max_ variability are ENR feed interactions, which occur when animals are not fasted sufficiently before dosing or when water is withheld prior to drug administration to minimize variations in the stomach (proventriculus and gizzard) emptying or the degree of ENR dilution (28).

The ENR-HCl T_max_ value found in this study (4.00 h) (Table 1) is in the range of the ENR-HCl dihydrate value reported by Gutierrez et al. (20) (3.10 h) and by Sumano et al. (27) (3.0–5.0 h, for generic ENR pharmaceutical preparations). However, they are slightly higher than those reported by Anadón et al. (14) (1.64 h) and by Gberindyer et al. (16) (1.0–2.0 h for three different brands), all broiler chickens treated orally. Furthermore, the outcomes of this study demonstrate that CIP reached C_max_ at 3.44 h after a single oral administration of the ENR-HCl product to broiler chickens.

Similar to previous studies (3, 6, 18), the biotransformation of ENR into its main metabolite CIP was low (4.91%), which indicates that, in broiler chickens, the product formulation containing ENR-HCl has a low level of ENR biotransformation.

Our findings indicated mean residence times (MRTs) of 10.60 ± 0.32 h and 10.59 ± 0.42 h for ENR-HCl and CIP, respectively (Table 1), which are similar to those values reported for ENR-HCl in turkeys (3) and broiler chickens (20). However, these values are higher than those reported for ENR (4–6 h) by Gberindyer et al. (16) and Aguilera et al. (30) and smaller than those (13–16 h) reported by Anadón et al. (14), Grabowski et al. (31) and Silva et al. (2). The half-life time of the elimination phase (t_1/2_ e) parameters (24.76 and 17.46 h for ENR-HCl and CIP, respectively) indicated a slow elimination rate. Contributing to this slow elimination is the fact that chickens have a highly developed biliary excretion mechanism, and thus, the drug is transferred along with the bile to the small intestine, where it is reabsorbed and participates in the enterohepatic cycle (32).

MIC values, determined for 10 bacterial strains responsible for inducing common clinical diseases in broiler chickens, were between 0.004 and 16 μg/mL. Two bacterial strains (*S*. ser. Gallinarum and *Avibacterium paragallinarum*) showed high resistance to the ENR-HCl product formulation. For the other eight susceptible strains (*Mycoplasma gallisepticum, Mycoplasma synoviae, Escherichia coli, Clostridium perfringens, Pseudomonas aeruginosa, S*. ser. Enteritidis, *S*. ser. Pullorum, and *S*. ser. Typhimurium), the MIC values were ≤ 0.5 μg/mL (Table 2).

The starting point of this research was to study the efficacy of ENR-HCl present in a powdered veterinary pharmaceutical product that is easily dissolved in drinking water to obtain more knowledge about this active substance. Thus, considering that only a few studies have reported on the difference in efficacy of this active substance with its parent compound (ENR base), the purpose of this research was to investigate this little-explored active substance so far.

Then, considering the PK/PD predictive indices of *in vivo* efficacy (AUC_0−24_/MIC and C_max_/MIC_90_ ratios) obtained in the present study (Table 2), it can be concluded that the veterinary pharmaceutical formulation containing ENR-HCl, administered orally (“*gavage*”) at a single dose (equivalent to 10 mg ENR base/kg bw), would be effective against *C. perfringens* (Gram-positive), *E. coli* and *S*. ser. Enteritidis (Gram-negative) and *M. gallisepticum* (mycoplasma) bacteria with MIC_90_ values lower than or equal to 0.125 μg/mL. Monte Carlo simulations were used to determine the probability of target attainment (PTA) for the PDIs (PK/PD index) of the ENR-HCl pharmaceutical product intended to treat bacterial strains that induce common clinical diseases in broiler chickens and that showed different MIC_90_ values (ranging from 0.004 to 16 μg/mL). The probability of attainment of the PDT (Target value of the PK/PD index) as a function of the distribution of MIC_90_ for the targeted bacteria is shown in Figure 2. The dose tested (10 mg/kg bw) has the probability to reach the PTA of 100% for *E. coli, M. gallisepticum*, and *S*. ser. Enteritidis strains with MIC_90_ values ≥0.06, 0.1 and 0.06 μg/mL, respectively. In relation to *C. perfringens* strains the probability to reach the PTA of ≥98.26% was for strains with MIC_90_ ≥ 0.12. It is important to recognize that to truly understand the relationship between antimicrobial exposure and the response, these findings must be considered in an integrated manner. The MIC_90_ value is used as an *in vitro* reference value to predict the antimicrobial efficacy and potency of a drug.

Overall, ENR-HCl has a wide margin of safety in broiler chickens. ENR-HCl was rapidly absorbed from the gastrointestinal tract, reaching peak blood levels quickly after oral ingestion. The long elimination half-life of ENR-HCl observed confers a significant post-antibiotic effect, which allows the ENR-HCl product formulation studied to be administered via an intermittent dose method in drinking water and takes advantage of its concentration-dependent killing, thus preventing the emergence of bacterial resistance (33).

In the European Union, ENR cannot be used for the treatment of Salmonella (34); however, in some countries, this practice is still carried out, and many formulations containing ENR are currently commercialized and are administered for Salmonella treatment. Therefore, to better understand the efficacy of ENR-HCl *in vitro* to combat these strains, the data results from this study demonstrate that only one (*S*. ser. Enteritidis) of the four types tested noticeably responded to the treatment (Table 2). The European Union regulatory agencies (13) evaluating cases of the use of FQs between 2011 and 2012 concluded that in 15 of 26 countries evaluated, the average antimicrobial consumption was lower or much lower in food-producing animals than in humans, and they observed associations between antimicrobial consumption and resistance prevalence for the selected bacterium–antimicrobial combinations that were analyzed in animals, in humans and from animals to humans. For *Salmonella* spp., this association was less clear, which underlies the fact that epidemiological resistance is complex and is influenced by many factors aside from the use of a particular class of antimicrobials, such as co-selection and clonal spread. The use of critically important antimicrobials (CIAs), such as ENR, and their importance in human medicine should be limited to cases where no other alternative is available (35).

Despite advances in the knowledge of pharmacokinetics and pharmacodynamics, there are still some challenges, especially in relation to the differences between species, genetic strain, sex, and age. The intra- and inter-species particularities are explained by several reasons, such as anatomical, physiological, and behavioral differences (32). There is also evidence that the drug formulation of veterinary medicine generates relevant effects on the pharmacokinetics and tissue depletion of a drug (36), and the effectiveness of a drug is dependent on its route of administration and metabolic pattern (37). Consequently, the development of a veterinary medicinal product requires the evaluation of its profile in the target species.

## Conclusion

The ENR-HCl after the administration of the veterinary product formulation evaluated here exhibited pharmacokinetic parameters comparable to those reported for ENR medicinal products available on the market, being an improved antimicrobial product of ENR-HCl for reasons of water solubility. Additionally, considering the AUC_0−24_/MIC_90_ and C_max_/MIC_90_ ratios obtained, the veterinary product formulation containing ENR-HCl, administered orally, at a single dose rate equivalent to 10 mg ENR base/kg bw, would be effective against *M. gallisepticum, E. coli, C. perfringens*, and *S*. ser. Enteritidis bacteria strains. The predictions obtained by the Monte Carlo simulations indicate that if the infectious bacteria *M. gallisepticum, E. coli*, and *S*. ser. Enteritidis have MIC_90_ values equal to or less than the values found in this research study for each of the individual bacteria, the dose equivalent to 10 mg ENR base/kg bw from the ENR-HCl pharmaceutical product meets 100% (TAR) of broiler chickens for C_max_/MIC_90_ = 10. For *C. perfringens*, the TAR is 98.26%. These results highlight the ENR-HCl veterinary product formulation as an interesting choice for farmers due to its ease and low handling cost. In continuity with this research study, Bonassa et al. (38, 39) established, in broiler chickens, a withdrawal period of 8 days for the oral administration of this veterinary product formulation.

## Data Availability Statement

The raw data supporting the conclusions of this article will be made available by the authors, without undue reservation.

## Ethics Statement

The animal study was reviewed and approved by Comissão de Ética no Uso de Animais (CEUA)/University of Campinas (Protocol number 3135-1).

## Author Contributions

KB and FR conceived and designed the study and wrote the manuscript. KB involved in collecting all the data. MM provided support to conduct the work with the broiler chickens. RS carried out the laboratory analysis. ME provided the laboratory conditions for the laboratory analysis. RM provided support with data interpretation. KB, AA, and FR reviewed and approved the manuscript. All authors contributed to the article and approved the submitted version.

## Conflict of Interest

MM was employed by company AGRIAS Pesquisa & Desenvolvimento S.A.R.C. no Agronegócio Ltda., Amparo, Brazil. The remaining authors declare that the research was conducted in the absence of any commercial or financial relationships that could be construed as a potential conflict of interest.
